# Case Report: Sacrococcygeal teratoma with malignant transformation into mixed adenocarcinoma and squamous cell carcinoma following TNF-α inhibitor therapy for ankylosing spondylitis

**DOI:** 10.3389/fonc.2026.1969865

**Published:** 2026-10-21

**Authors:** Peiyi Luo, Miaomiao Zhang, Ruxin Zhang, Junjian Deng, Yongfa Zheng

**Affiliations:** Center of Oncology, Renmin Hospital of Wuhan University, Wuhan, China

**Keywords:** adenocarcinoma, case report, sacrococcygeal teratoma, squamous cell carcinoma, TNF-α inhibitor

## Abstract

Adult sacrococcygeal teratoma (SCT) with malignant transformation is exceptionally rare, and its occurrence during long-term tumor necrosis factor-α (TNF-α) inhibitor therapy has never been reported. This case firstly links prolonged TNF-α blockade—used for ankylosing spondylitis—with aggressive biphenotypic (i.e. adenocarcinoma and squamous cell carcinoma (SCC)) transformation of congenital SCT. Serial biopsies over time enabled dynamic tracking of histological changes from adenocarcinoma toward SCC, a phenomenon not previously documented in malignant SCT. The concurrent TP53 and KRAS G12D mutations further distinguish this case and suggest a potential targeted therapeutic avenue. A 31-year-old woman with two prior resections of gluteal masses and six-year TNF-α inhibitor exposure presented with a recurrent gluteal mass. Imaging revealed a pelvic–sacral mass and a gluteal subcutaneous soft tissue mass. Biopsy of the pelvic tumour only described intestinal-type adenocarcinoma; however, subsequent gluteal biopsy disclosed mixed components of 65% SCC and 35% adenocarcinoma. The origin diagnosis was malignant SCT with adeno-squamous transformation, IV stage with pelvic and gluteal metastases. Unfortunately, the patient successively developed local recurrence at the sacrococcygeal region and widespread metastases involving liver, inguinal lymph nodes, bone, lungs, and kidneys, although multidisciplinary Interventions were administered including resection, chemotherapy and radiotherapy. Clinicians should learn two key lessons from this case. First, it is imperative to sustain vigilance for malignant transformation in patients with a history of recurrent SCT, and regular follow-up is strongly advised. Second, although current meta-analyses demonstrate no statistically significant association between TNF-α inhibitor exposure and overall malignancy risk, the physiological role of TNF-α in immune surveillance, together with relevant clinical guidelines, supports discontinuation of these agents upon diagnosis of a solid malignancy. In this case, given the aggressive tumor behavior and the availability of alternative agents for ankylosing spondylitis, we elected to pause TNF-α blockade as a precautionary, guideline-informed clinical judgment.

## 1 Introduction

Teratomas are germ-cell tumors derived from pluripotent cells, containing tissues representative of two or three germ layers (i.e. ectoderm, mesoderm and endoderm). Mature teratomas are well-differentiated benign lesions, typically presenting as unilocular cystic masses (also named dermoid cysts) filled with hair, teeth, sebaceous material, bone fragments, or neural tissue. Malignant transformation—defined as the development of a non-germ-cell malignancy from any mature somatic component within a mature teratoma—is a rare complication, representing approximately 1%–2% of all mature teratomas. Ovarian teratoma accounts for the majority of cases, although midline locations such as the mediastinum and sacrococcygeal region have also been reported (1, 2). Histologically, squamous cell carcinoma (SCC) predominates, representing >75%–80% of cases, followed by adenocarcinoma (i.e. intestinal and mucinous types), sarcomas (i.e. leiomyosarcoma, chondrosarcoma and rhabdomyosarcoma), and exceptionally, malignant melanoma, neuroendocrine carcinoma, as well as papillary thyroid carcinoma (3, 4).

Sacrococcygeal teratoma (SCT) is primarily a pediatric tumor; adult presentation is exceedingly rare, with an incidence of 1 in 63,000 to 1 in 40,000 and a female-to-male ratio of 3:1 (4). A meta-analysis of 1,516 SCT cases reported pooled recurrence rates of 3% for mature tissue and 5% for malignant components; among 102 recurrences, 56% were malignant, predominantly yolk sac tumors (YST), with most relapses occurring within 1–6 years after initial surgery, though late recurrences up to 20 years have been noted (5). However, that study was confined to the pediatric population, and its malignant recurrences were predominantly YST—likely representing microscopic foci overlooked at initial pathological evaluation, which do not strictly meet the criteria for somatic malignant transformation. In adults, SCT with malignant transformation is extremely rare, with only several case reports in the literature and no systematic reviews or clinical practice guidelines available. A 45-year-old woman developed adenocarcinomatous transformation of a SCT 13 years after prior pelvic surgery, a finding that underscores the decisive prognostic significance of radical resection (i.e. R0 resection) (6). A 62-year-old woman whose presacral tumor transformed into adenocarcinoma after 10-year follow-up underwent combined abdominoperineal resection with sacrectomy at the S5 level; Despite developing inguinal lymph node metastases 7 months postoperatively, she underwent lymphadenectomy and remained recurrence-free for 21 months, demonstrating that radical surgery with aggressive management of metastatic deposits can still achieve favorable long-term survival (7). A 40-year-old woman with congenital SCT, who presented with rapid enlargement over 3 months and fever, received puncture aspiration but followed by early recurrence. Complete resection ultimately verified that malignant transformation to SCC happened in the SCT, revealing the potential risk of SCT progression to malignant phenotype and the need for vigilance when any sudden enlargement occurs (8).

Above-mentioned cases only involved malignant transformation of one single histological component within SCT. Here, we report the first case of malignant recurrence of adult SCT in a patient on long-term tumor necrosis factor-α (TNF-α) inhibitor therapy, with histopathology demonstrating biphenotypic differentiation—both squamous and adenocarcinomatous components. The patient underwent multiple lines of treatment, including retroperitoneal mass resection, FOLFOX (i.e. 5-fluorouracil plus oxaliplatin) chemotherapy, TP (i.e. paclitaxel polymeric micelle plus carboplatin) chemotherapy, sacrococcygeal tumor resection with abdominal lymphadenectomy, radiotherapy to inguinal and hepatic metastases, irinotecan chemotherapy, and paclitaxel chemotherapy plus anlotinib targeted therapy. Despite these aggressive interventions, the disease showed relentless progression, with eventual dissemination to the lungs, liver, kidneys, and bones. This case carries several important clinical implications. First, although large-scale meta-analyses have not established a statistically significant association between TNF-α inhibitor exposure and overall malignancy risk, TNF-α is physiologically critical in tumor immune surveillance. Sustained TNF-α blockade could theoretically impair immune-mediated clearance of malignant cells—a concern reflected in French clinical practice guidelines endorsed by the French National Authority for Health (HAS) that advise discontinuation of TNF-α antagonists upon diagnosis of a solid malignancy (9). Second, serial biopsies of both primary and metastatic lesions enabled dynamic tracking of histological shifts from adenocarcinoma to SCC, facilitating real-time clinical decision-making. Third, genomic profiling revealed concurrent TP53 and KRAS G12D mutations, the latter of which may offer a rationale for targeted therapeutic intervention in the future.

## 2 Case description

A 31-year-old woman had undergone two prior excisions of gluteal masses at approximately 1 and 20 years of age, with unknown pathological outcomes. She had a history of ankylosing spondylitis, and had received etanercept from 2019 to 2024. At that time she had developed uveitis, and thereafter began to use certolizumab pegol and adalimumab intermittently. She denied other significant medical, family and psycho-social history. On June 10, 2025, she sought medical attention at a local hospital for a painless gluteal mass she had noticed over the preceding week. Physical examination described a 3-cm mass in the right buttock without erythema, ulceration, or discharge. Computed tomography (CT) showed a cystic-solid mass in the sacral and pelvic region, suggestive of a dermoid cyst, along with a nodule in the basal segment of the right lower lobe and multiple small solid nodules in both lungs. On June 16, 2025, laparoscopic retroperitoneal mass resection was performed. Postoperative pathology of the pelvic mass revealed mucinous adenocarcinoma, presumed to be of gastrointestinal origin. Immunohistochemistry (IHC) described Villin (+), CDX-2 (+), CK20 (+), SATB2 (+), CK7 (+), Ki67 (+, 80%). A follow-up pelvic CT on June 20, 2025 showed post-resection changes and a solid density in the right subcutaneous sacral region (**Figure 1**).

**Figure 1:**
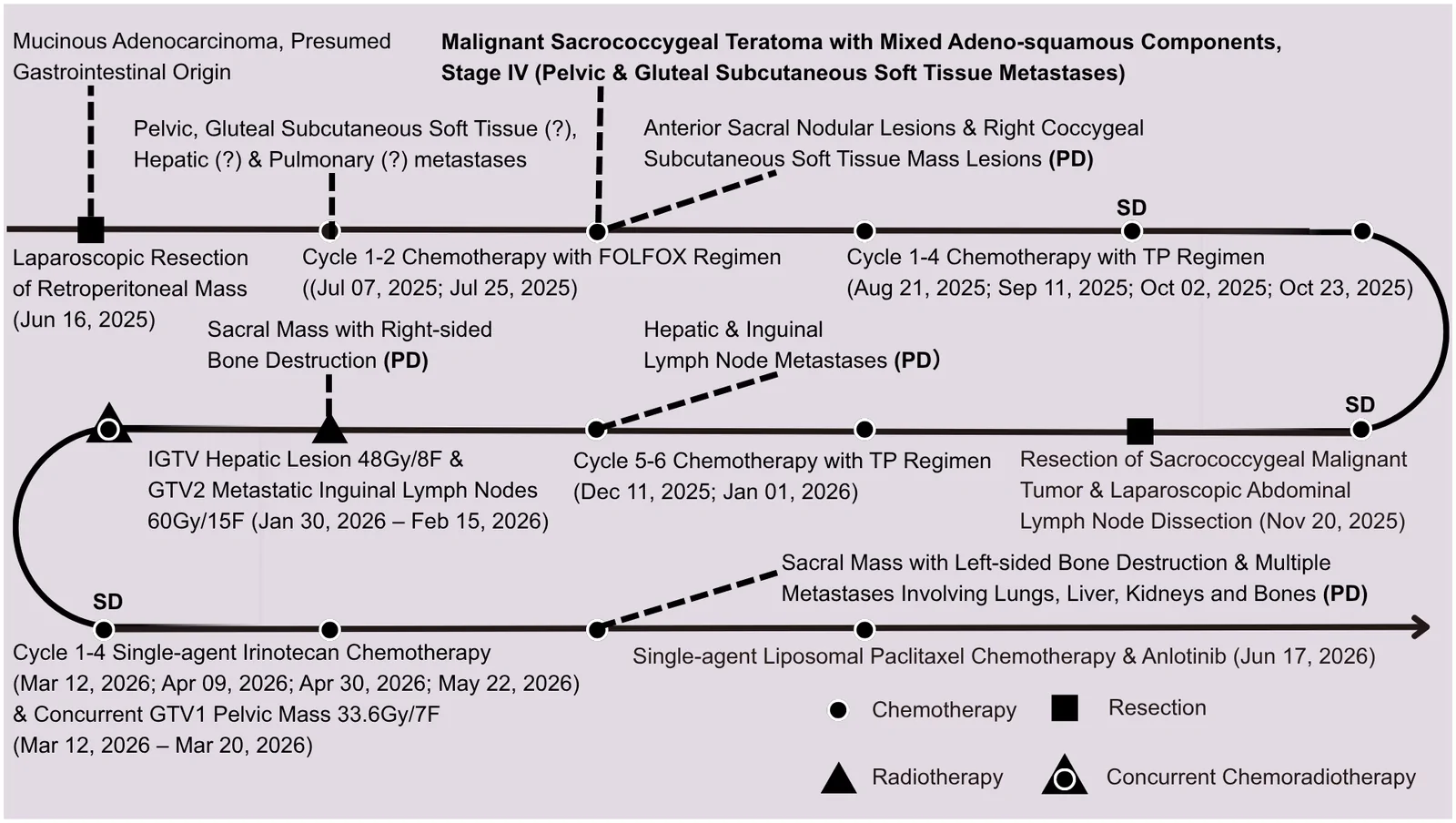
Clinical timeline. The timeline summarizes diagnostic updates, sequential treatments (surgery, chemotherapy, radiotherapy, targeted therapy) and corresponding efficacy evaluations from June 2025 to June 2026 in a patient with malignant SCT (Mixed adeno-squamous components, stage IV).

She was then transferred to our hospital, and ^68^Ga-FAPI PET-CT on June 26, 2025 revealed soft-tissue lesions in the pelvic mesentery and presacral space, suggestive of metastases; a subcutaneous mass in the right gluteal area, consistent with local recurrence; and multiple pulmonary nodules, some of which were suspicious for metastasis. Postoperative recurrence was confirmed, and the tumor was considered to be of gastrointestinal adenocarcinoma origin. Therefore, the first cycle of FOLFOX chemotherapy was administered on July 7, 2025. On July 9, 2025, pathological review of the pelvic mass at our hospital revealed intestinal-type adenocarcinoma, with an IHC profile identical to that of the initial specimen, but suggestive of SCT with malignant transformation. Supplementary IHC showed PD-L1 (CPS = 2) and Claudin18.2 (−). Genetic testing demonstrated microsatellite-stable (MSS) status, with TP53 and KRAS G12D mutations. The second FOLFOX chemotherapy was administered on July 25, 2025. On July 28, 2025, electronic gastroscopy and colonoscopy disclosed chronic non-atrophic gastritis with erosions, proctitis, and hemorrhoids, effectively excluding a primary gastrointestinal origin of the tumor. CT showed: (i) postoperative changes in the pelvic floor; (ii) multiple patchy and nodular opacities in the right gluteal subcutaneous tissue; (iii) a nodular lesion anterior to the sacrum, approximately 2.3 cm in diameter, and a soft-tissue mass lateral to the coccyx measuring 4.5 × 4.0 cm in maximum cross-section, suggestive of local recurrence given the clinical history; (iv) multiple pulmonary nodules and patchy opacities, suspicious for metastasis; (v) a patchy enhancing lesion in hepatic segment S4 during the arterial phase and a weakly enhancing nodule in segment S6, also suggestive of metastasis. On August 18, 2025, gluteal ultrasonography revealed a mixed echogenic mass measuring approximately 5.3 × 3.7 cm in the right buttock, and ultrasound-guided puncture biopsy was performed. Pathology of the gluteal soft tissue showed poorly differentiated carcinoma, comprising approximately 65% poorly differentiated SCC and 35% low-to-moderately differentiated intestinal-type adenocarcinoma, consistent with malignant transformation of SCT. IHC describes Villin (+), CDX-2 (+), CK20 (+), SATB2 (+), P40 (+), CK5/6 (+), CK7 (+), Ki67 (+, 85%) (**Figure 2**). Synthesizing all findings, which excluded primary gastrointestinal adenocarcinoma, malignant transformation of a tailgut cyst (10), and sacral chordoma, a multidisciplinary team discussion (i.e. departments of Gynecology, Gastrointestinal Surgery, Rheumatology and Immunology, Oncology, and Pathology) reached a final diagnosis: SCT with malignant transformation into mixed adenocarcinoma and SCC, stage IV with pelvic and right gluteal subcutaneous soft-tissue metastases.

**Figure 2:**
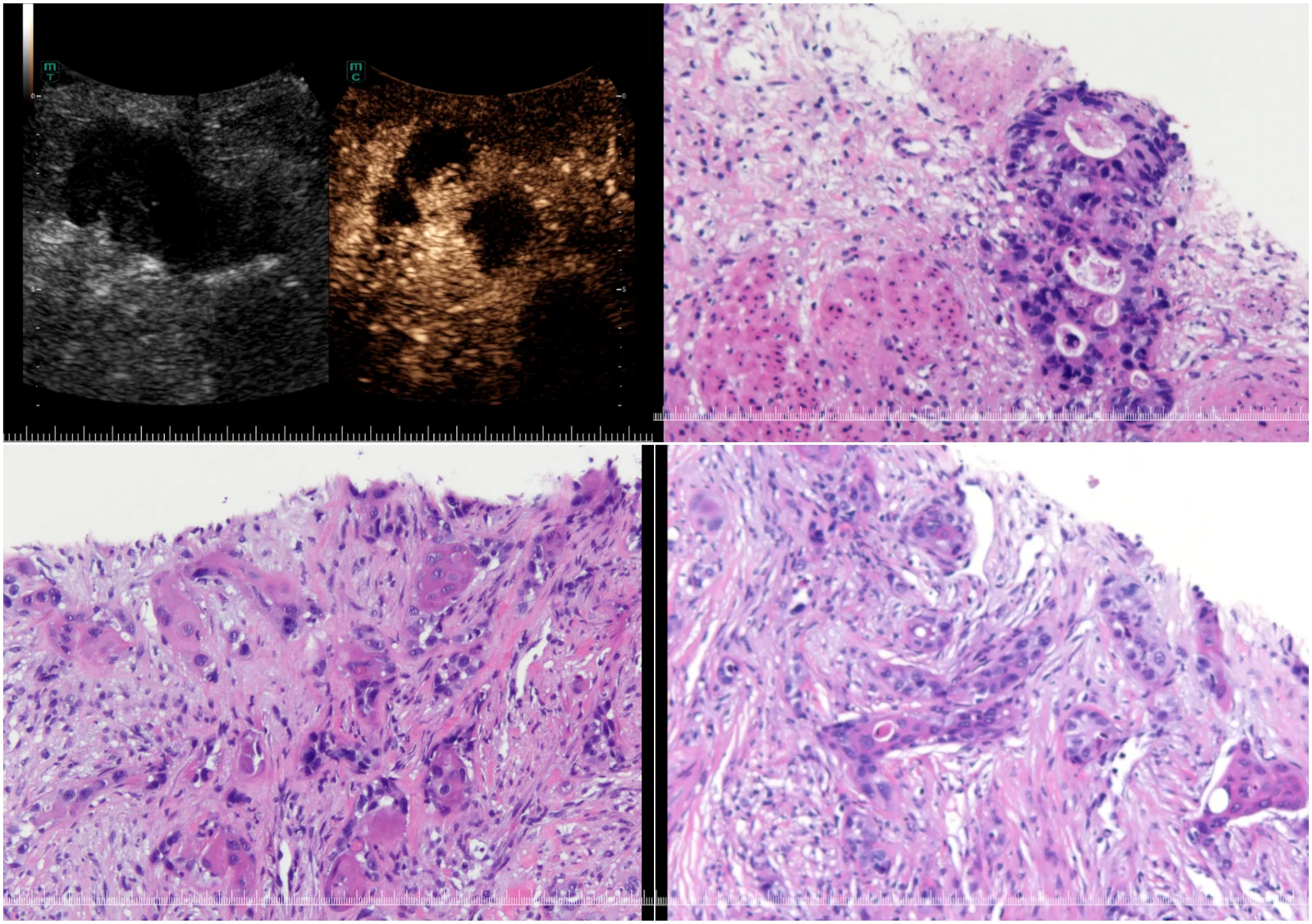
Gluteal Ultrasonography and Corresponding H&E-stained Histological Sections after Puncture Biopsy on August 18, 2025.

Given that progressive disease (PD) was determined according to the Response Evaluation Criteria in Solid Tumors (RECIST) (11) at the first treatment-response assessment, and the tumor was considered to be predominantly SCC, four cycles of TP chemotherapy were administered on August 21, September 11, October 2, and October 23, 2025. Interim CT on October 2, 2025, demonstrated stable disease (SD). Another CT on November 13, 2025, again showed SD. On November 20, 2025, sacrococcygeal tumor resection combined with laparoscopic abdominal lymphadenectomy was performed. Postoperative pathology of the sacrococcygeal tumor revealed poorly differentiated carcinoma, partially intestinal-type adenocarcinoma and partially SCC, consistent with malignant transformation of SCT, with intratumoral vascular invasion and perineural infiltration identified. IHC describes Villin (+), CDX-2 (+), SATB2 (+), P40 (+), CK7 (+), Ki67 (+, 80%). On December 10, 2025, CT demonstrated postoperative changes in the pelvic floor and sacrococcygeal region, with the previously noted right-sided sacrococcygeal mass no longer visible; multiple pulmonary nodules, with a subpleural nodule in the right lower lobe suggestive of metastasis; and a slightly hypodense nodule near the capsule of the right hepatic lobe, suspicious for metastasis. The 5th and 6th cycles of TP chemotherapy were administered on December 11, 2025, and January 1, 2026, respectively.

On January 23, 2026, CT demonstrated: (i) a subpleural nodule in the right lower lobe showing slight enlargement compared with previous imaging; (ii) a slightly hypodense nodule near the capsule of the right hepatic lobe, unchanged from prior imaging; and a new hypodense lesion in the left hepatic lobe, more conspicuous compared with previous imaging; (iii) small bilateral inguinal lymph nodes, some of which had increased in size. The remainder of the CT findings was largely unchanged. On the same day, ultrasound-guided puncture biopsy of the right inguinal region was performed. Pathology of the inguinal lymph node revealed metastatic carcinoma, consistent with SCC metastasis. IHC describes Villin (-), CDX-2 (-), CK20 (-), SATB2 (-), P40 (+), CK5/6 (+), Ki67 (+, 60%) (**Table 1**). On January 26, 2026, magnetic resonance imaging (MRI) of the upper abdomen demonstrated an abnormally enhancing lesion in the right hepatic lobe and an abnormal paraspinal lesion in the right lower lobe of the lung. PD was determined. From January 30 to February 15, 2026, radiotherapy was delivered, with an internal gross tumor volume (IGTV) of 48 Gy in 8 fractions to the hepatic lesion and a gross tumor volume (GTV) of 60 Gy in 15 fractions to the metastatic inguinal lymph nodes. On March 10, 2026, CT showed: (i) multiple bilateral pulmonary nodules, some with cavitations; (ii) the slightly hypodense nodule near the capsule of the right hepatic lobe had decreased in size; (iii) right inguinal lymph nodes had enlarged compared with previous imaging; (iv) a new osteolytic lesion in the right sacrum with an associated soft-tissue mass, representing a new finding; (v) new hypodense lesions anterior and to the left of the sacrum. The remainder of the CT findings was largely unchanged. The patient had been exposed to 5-fluorouracil (i.e. thymidylate synthase inhibitor (12)), oxaliplatin (i.e. DNA crosslinker (13)), paclitaxel (i.e. microtubule stabilizer (14)), and carboplatin (i.e. DNA crosslinker (13)), however, persistent PD determined by CT suggested the possibility of acquired resistance to them. Irinotecan, a topoisomerase I inhibitor, acts through a distinct mechanism without theoretical cross-resistance (15, 16) and demonstrates activity against both adenocarcinomatous (17, 18) and squamous components (19, 20), supporting its empirical use in this biphenotypic malignancy. Therefore, the patient received four cycles of irinotecan monotherapy as the third-line treatment on March 12, April 9, April 30, and May 22, 2026. During this period, radiotherapy to the pelvic mass (GTV 33.6 Gy in 7 fractions) was administered from March 12 to March 20, 2026. After two cycles of irinotecan, CT demonstrated SD.

**Table 1:** Comparative Immunohistochemical Profiles of Primary and Metastatic Lesions at Serial Time Points.

Marker	Pelvic Region (Jun 16, 2025)	Pelvic Region (Jul 09, 2025)	Gluteal Soft Tissue (Aug 18, 2025)	Sacrococcygeal Region (Nov 20, 2025)	Inguinal Lymph Node (Jan 23, 2026)	Liver (Jun 16, 2026)
Villin	(+)	(+)	(+)	(+)	(-)	(-)
CDX-2	(+)	(+)	(+)	(+)	(-)	(-)
CK20	(+)	(+)	(+)	NP	(-)	(-)
SATB2	(+)	(+)	(+)	(+)	(-)	NP
P40	NP	NP	(+)	(+)	(+)	(+)
CK5/6	NP	NP	(+)	NP	(+)	(+)
CK7	(+)	(+)	(+)	(+)	NP	(+)
Ki67	80%	80%	85%	80%	60%	70%

NP, Not Performed.

On June 12, 2026, the patient reported pain and abscess formation at the surgical incision site. CT illustrated: (i) multiple bilateral pulmonary nodules, some with cavitations, with increased solid components and slight enlargement in some lesions compared with prior imaging; (ii) multiple slightly hypodense lesions in the liver, increased in both number and size; (iii) a new osteolytic lesion in the left sacrum with an associated soft-tissue mass; (iv) a new foci of subcutaneous emphysema in the gluteal region; (v) new abnormally enhancing lesions in both kidneys, suspicious for metastasis. The remainder of the findings was largely unchanged (**Figure 3**). PD was determined. A multidisciplinary team discussion concluded that the patient, with widespread metastases to the lungs, liver, kidneys, and bones, was not a candidate for surgical intervention, and conservative medical management was recommended. In accordance with the consensus of the multidisciplinary team, and given concern that TNF-α inhibitors may pose a risk of tumor progression, these agents were temporarily discontinued on June 15, 2026. Supportive care was initiated, comprising analgesia, empirical antibiotic therapy with cefoperazone-sulbactam, and wound dressing of the gluteal incision site. Acid-fast bacilli staining of wound secretions and the T-cell spot test for tuberculosis infection both yielded negative results, ruling out tuberculosis. Consequently, the acute local infection was controlled, as evidenced by decreased wound discharge, subsiding erythema, and regressed subcutaneous emphysema. On June 16, 2026, ultrasound-guided liver biopsy was performed. Pathology of the liver lesion revealed malignant tumor with necrosis, consistent with metastatic SCC. IHC describes Villin (-), CDX-2 (-), CK20 (-), P40 (+), CK5/6 (+), CK7 (+), Ki67 (+, 70%). Given the patient’s failure of multiple prior lines of therapy, later-line salvage therapy was considered. Anlotinib, a multi-target tyrosine kinase inhibitor (TKI), exerts dual antitumor effects by inhibiting angiogenesis via VEGFR, PDGFR, and FGFR, and directly suppressing tumor cell proliferation (21). It has been approved in China for the later-line treatment of advanced soft tissue sarcoma that has progressed or recurred after prior anthracycline-based chemotherapy (22). Therefore, the patient received paclitaxel liposome chemotherapy combined with anlotinib targeted therapy on June 17, 2026.

**Figure 3:**
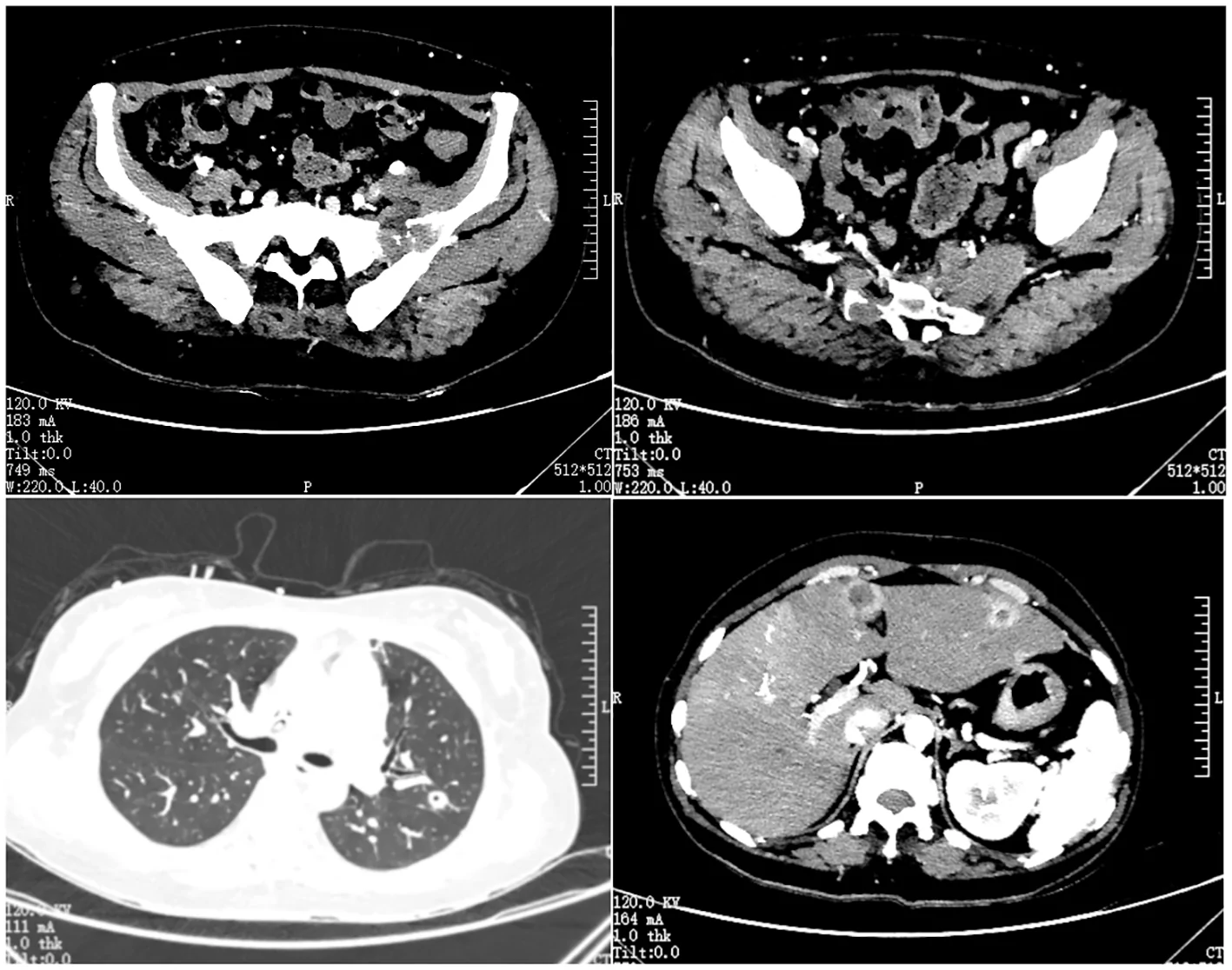
Axial CT Scans On June 12, 2026. These images illustrate osteolytic lesions in both sides of sacra with associated soft-tissue masses, lung and liver metastases.

## 3 Discussion

Retrospective review of this case reveals that the patient had undergone two resections of gluteal masses at approximately 1 and 20 years of age, neither of which yielded a definitive pathological diagnosis or was followed by regular follow-up, thereby laying a latent risk for malignant transformation. In patients with a history of recurrent SCT, a further recurrence is more likely to be malignant, and once malignant transformation occurs, the prognosis becomes extremely poor (23). This underscores that for any recurrent mass of uncertain identity, definitive biopsy and regular follow-up are essential measures to prevent malignancy.

The patient had been on long-term TNF-α inhibitor therapy for ankylosing spondylitis. The decision to suspend TNF-α antagonist therapy in this patient was reached through a multidisciplinary assessment based on integrative considerations. At the mechanistic level, TNF-α is recognized as a core component of protective tumour immune surveillance, capable of promoting immune recognition to mediate tumour cell apoptosis (24, 25). Sustained pharmacological inhibition of TNF-α could, in theory, attenuate the host’s capacity to eliminate malignant cells, raising a legitimate biological concern. However, a study encompassing 45 interventional and 10 observational studies with over 57,000 patients demonstrated no statistically significant association between TNF-α inhibitor exposure and overall malignancy risk (26). This finding was corroborated by another study focusing specifically on psoriasis patients; notably, its subgroup analysis revealed an increased risk of non-melanoma skin cancer among psoriasis patients receiving TNF-α inhibitors, with a particularly pronounced risk for SCC (27). Despite the lack of epidemiological evidence for causality, clinical practice guidelines have addressed this scenario from a management perspective. The French recommendations for using TNF-α antagonists endorsed by HAS (9) explicitly state: ‘If a solid malignancy is diagnosed during TNF-α antagonist therapy, the TNF-α antagonist should be stopped.’ This expert opinion-based recommendation reflects a precautionary principle that preserving immune surveillance takes precedence during active malignancy, irrespective of whether the drug played a causative role. Integrating these three dimensions—biological plausibility, epidemiological neutrality, and guideline-based management—we considered TNF-α antagonist discontinuation a prudent clinical judgment in this specific context. Several case-specific factors further supported this decision: (i) the aggressive biphenotypic malignant transformation of SCT in this patient exhibited relentless progression despite multimodal interventions, creating an urgent clinical context in which any potentially modifiable factor that could theoretically impair anti-tumor immunity warranted meticulous avoidance; (ii) the narrow therapeutic window inherent to this malignancy demanded that all management decisions be prioritized toward oncological control; and (iii) the patient’s ankylosing spondylitis, being a chronic but slowly progressive condition, could be temporarily managed with alternative agents (e.g. NSAIDs or corticosteroids) during the short-term oncological treatment phase, minimizing the risk of rheumatological flare and providing a practical buffer for biological therapy suspension. We emphasize that this represents a case-specific, guideline-informed clinical judgment rather than a universally applicable protocol. We do not advocate for routine discontinuation of TNF-α inhibitors in all cancer patients; rather, we suggest that in the setting of rapidly progressive, treatment-refractory malignancies with limited therapeutic options, a multidisciplinary re-evaluation of concurrent immunomodulatory therapies is reasonable, with explicit recognition of the current evidence limitations.

This case demonstrates, for the first time, spatiotemporal histological heterogeneity—a progressive shift from adenocarcinoma to squamous predominance—in the setting of malignant SCT. However, in the absence of molecular clonality analysis and given the sampling from distinct anatomical sites, we cannot definitively attribute this phenomenon to any specific mechanism. The observed heterogeneity could arise from: (i) multiclonal origin, where adenocarcinoma and squamous components derive from two independent clones that metastasized separately (28, 29); (ii) clonal selection under therapeutic pressure, where pre-existing chemotherapy-sensitive adenocarcinoma clones were eliminated, allowing resistant squamous clones to expand (30, 31); or (iii) lineage plasticity, where a single adenocarcinoma clone underwent transdifferentiation into squamous phenotype, predominantly driven by transcriptional and epigenetic reprogramming rather than genetic mutations (32, 33). In this case, serial biopsies dynamically captured histological heterogeneity, guided flexible therapeutic decisions, and highlighted the clinical value of real-time pathology-based adaptive therapy. We acknowledge that molecular clonality analysis which would be essential to distinguish among the above mentioned mechanisms was not performed. This limitation was primarily due to financial constraints and the limited clinical necessity of such analysis (i.e. the initial sequencing had already identified the driver mutations to guide treatment). Nevertheless, we fully recognize that elucidating the clonal architecture underlying histological shifts holds substantial scientific value, and future investigations with multi-region sequencing or single-cell genomic approaches would be warranted to resolve this biological question.

The patient harbored a KRAS G12D mutation. According to the Insight database, 42 KRAS G12D-targeted agents have entered clinical development globally, with the 6 most advanced candidates already in Phase III trials, including DN022150 and setidegrasib. Unlike setidegrasib, which acts as a KRAS G12D degrader, DN022150 specifically binds the Switch-II pocket of the mutant KRAS G12D protein, engaging both the GDP-bound (OFF) and GTP-bound (ON) states, thereby inhibiting KRAS-mediated signal transduction and downstream RAS–MAPK pathway activation to exert antitumor effects (34). Given that the patient has failed multiple lines of therapy, actively pursuing enrollment in a clinical trial of a KRAS G12D inhibitor represents the most promising therapeutic strategy at present. Importantly, the TP53 mutation identified in the tumor genome of this patient raises the critical question of whether it represents a somatic alteration or a germline variant. Li-Fraumeni syndrome (LFS), caused by germline pathogenic variants in the TP53 gene, is characterized by early-onset malignancies, multiple primary tumors, and a lifetime cancer risk approaching 100% in females (35). The classic LFS diagnostic criteria include a proband with sarcoma before age 45, a first-degree relative with cancer before age 45, and an additional first- or second-degree relative with cancer before age 45 or sarcoma at any age (36). NCCN guidelines suggest that in individuals with cancer with a P/LP TP53 variant identified on tumour only genomic testing, germline testing should be considered for those who have family tumour history, those diagnosed age <30 with any cancer or those with clinical scenario not meeting these criteria but warranting germline evaluation per clinician discretion (37). Therefore, although the patient denied family tumour history, the presence of a pathogenic TP53 variant warrants consideration of germline testing. Unfortunately, germline testing was not performed, as the genomic profiling was conducted on tumor tissue for therapeutic decision-making, and the patient was not referred for formal genetic counseling. We acknowledge this as a limitation of the case and strongly recommend that genetic counseling and germline TP53 analysis be pursued to exclude LFS, which would have profound implications for the patient’s future cancer surveillance and for the cancer risk assessment of family members.

This case also provides preliminary clinical experience with anlotinib as a salvage therapy in refractory malignant SCT (38). However, the anti-VEGFR/FGFR/PDGFR mechanisms of anlotinib may interfere with angiogenesis and fibroblast function (39), and the antitumor benefit must be carefully weighed against the risk of wound complications. During anlotinib treatment, close monitoring and integrated wound management are warranted.

Finally, we emphasize that as a single-case design, our findings are inherently hypothesis-generating rather than generalizable, and treatment decisions were individualized via multidisciplinary consensus rather than predefined protocols, reflecting the real-world complexity of managing such a rare and aggressive malignancy.

## Patient perspective

As a young woman diagnosed with a rare tumor, my journey over the past year has been challenging. What started as a small buttock lump led to multiple surgeries and several different chemotherapy regimens, each bringing its own side effects and uncertainties. When I was told that the cancer had spread to my liver, lungs, and bones despite all the treatments, I felt deeply frustrated and exhausted. However, the medical team never gave up on me. They explained every step clearly, arranged multidisciplinary consultations, and always listened to my concerns. The decision to try a new targeted therapy gave me renewed hope, even though I knew the chances were uncertain. I am grateful for the compassion and professionalism of every doctor and nurse who cared for me. I have agreed to share my story because I hope it may help other patients facing similar difficult battles, and perhaps contribute to better understanding of this rare disease.

## Data Availability

The original contributions presented in the study are included in the article/supplementary material. Further inquiries can be directed to the corresponding author/s.
